# How to eliminate hepatitis C between people who inject drugs in community services and prisons in Catalonia

**DOI:** 10.1186/s12954-025-01286-w

**Published:** 2025-07-31

**Authors:** Elena Yela, Daniel G. Abiétar, Rafael Clua-García

**Affiliations:** 1 Sant Esteve Sesrovires Prison Primary Care Team (Brians 1 Prison Centre), Catalan Health Institute, Barcelona, Spain; 2 Institut Universitari d’Investigació en Atenció Primària Jordi Gol i Gurina (IDIAPJGol), Barcelona, Spain; 3 South Metropolitan Research Support Unit Foundation, University Institute for Research in Primary Health Care Jordi Gol i Gurina (IDIAPJGol), El Prat de Llobregat, , Spain; 4 Network for Research on Chronicity, Primary Care, and Health Promotion (RICAPPS), Barcelona, Spain; 5 Manresa Faculty of Health Sciences, University of Vic– Central University of Catalonia, Manresa, Spain

**Keywords:** Hepatitis C, People who inject drugs, Direct-acting antivirals, Harm reduction

## Abstract

**Background:**

The Spanish National Health System, with devolved powers to autonomous communities such as Catalonia, faces significant challenges in controlling viral infections like hepatitis C (HCV) among vulnerable groups, particularly people who inject drugs (PWID), where prisons serve as crucial intervention sites. Catalonia’s health authorities have implemented strategies to combat HCV, including direct-acting antiviral (DAA) treatments and harm reduction programmes within both community and penitentiary settings. However, substantial barriers persist in achieving full treatment uptake and clearance among PWID subpopulations.

**Main body:**

This review aims to discuss the Catalonia’s current HCV programmes and explores intervention proposals needed to achieve WHO elimination targets. Catalonia has implemented a comprehensive HCV plan, particularly targeting PWID, that has proven effective through enhanced screening, universal treatment access, and harm reduction, though structural and social barriers remain due to fragmented health and social systems.

**Conclusion:**

Advancing towards HCV elimination requires strengthened inter-organisational coordination, integrated social and health services, simplified care pathways, enhanced screening, professional training, targeted research, measurable goals, culturally appropriate and participatory prevention strategies, and a comprehensive, people-centred approach. This is particularly important in prisons, where universal screening, adapted caring processes, harm reduction, and opioid substitution treatments (OST) are essential. Considering the social determinants of health perspective, it is essential that policies and programs are structured to reduce structural inequities and vulnerabilities, thereby promoting equity in both access to prevention, care, treatment, and health benefits across all population groups, particularly those most affected.

## Context of health policies for HCV prevention and control in Catalonia

The Spanish National Health System is a universal public healthcare system with its powers devolved to the autonomous communities, including Catalonia, while the Ministry of Health oversees overall coordination across regions. Despite an epidemiological transition towards a higher burden of non-communicable diseases, its challenges include the control of infections by viruses such as HCV and HIV [1] within vulnerable groups, especially drug users [2, 3]. Health services focused on disease prevention and health promotion are especially crucial in this context, since this population has to deal with self-care challenges and a notable lack of social and public health interventions in the community, as it is particularly noticeable, for example, in the prison-leaving process [4]. For this reason, among others, prisons offer a key intervention opportunity to reduce blood transmission infections linked to parenteral drug use [5–7] and, more specifically when dealing with HCV, prison programmes are key to global elimination programmes [7].

In 2015 the approval of the aforementioned health plan led to enhanced detection, treatment with DAA and the epidemiological monitoring of hepatitis C, as well as prevention campaigns for the general population [1]. The Department of Health of the Government of Catalonia, making use of its health competencies in community health services and prisons, implemented an HCV approach plan, paying special attention to PWID. Community services have intensified universal screening and single-step diagnosis for HCV while specialists have approached services for PWIDs, specifically in drug centres (DC) and harm reduction centres (HRC) [8, 9]. It focused on diagnosis and early treatment in primary care services and the referral to specialised services for HCV treatment, as well as on the intensification of community and penitentiary harm reduction programmes included in these centres, which are part of an extended drug attention network [3, 10]. When it comes to prisons, the approach is based on early detection through universal screening upon prisoner entrance and access to treatments under the same conditions as those of the general population [11].

It is well established that the prevalence of active HCV infection among PWID and incarcerated populations remains a major public health concern. Among PWIDs in Catalonia, the prevalence of active HCV infection is estimated at approximately 50% [12, 13] through screening in HRCs, where only 46% accept to undergo testing in previous studies [12]. However, in Catalan prisons, screening is ensured with a high acceptance rate of over 80% [5] the global prevalence of entering people is 2% [14].

Despite a strong deployment of prevention and control methods from specific health centres, such as drug dependence care units and harm reduction services in the community and prisons we detected difficulties in HCV clearance in more than half of the PWID diagnosed [15, 16]. We must therefore analyse the situation in certain subpopulations of PWID who present cultural or structural barriers to accessing HCV screening and treatment. It is also needed to consider intervention proposals more adapted to the different subpopulations in order to reach the goal of HCV elimination among PWID in Catalonia. This poses two questions: what is the current impact of the HCV prevention and treatment strategy in Catalonia? What proposals are required to achieve the WHO’s objectives? This paper addresses these two questions from a narrative review that examines the main results of the studies carried out in Catalonia and their recommendations for eliminating HCV.

## Situation of HCV infection among PWIDs

In our context the PWID demographic is the group that offers the main challenge to the elimination of HCV, given the persistently high prevalence. Among those screened in HRCs, active infection rates range from 42 to 55%. It is a significantly higher figure than among people who access DC, and the previously unknown diagnosis being one of 19% of cases [12, 13]. The prevalence of both HCV and HIV coinfection among PWIDs has improved in the last decade, with prevalence decreasing by 3.5% and 2.1%, respectively [17]. That said, the success of the programmes implemented in HRCs in Catalonia has been conditioned by low acceptance of screening, patient losses and early reinfections [12].

In this global improvement, women do not follow the same trend as men, since the prevalence of both HCV infection and coinfection has increased by 17.8% and 5.7% in Catalonia, respectively [17]. In the case of young PWIDs, between 2008 and 2019, the prevalence of HIV, HCV and HIV-HCV coinfection decreased significantly, especially among men, which reflects the positive impact of specialised harm reduction services [17].

The proportion of migrants PWIDs has increased in the last decade, new injectors amounting to 59% [18]. The important barriers for such people are reflected in a lower HCV screening coverage compared to natives, less knowledge about the infection and fewer opportunities for accessing treatment [8, 19].

Men who have sex with men (MSM) or transgender people are recognized as a vulnerable population, with a prevalence of HCV infection of 0.75% [20]. Specific recommendations have been added for this group in relation to screening and specialist referral in Catalonia by means of the HCV rapid detection test protocol for MSM and trans people of the Health Department [21], and special attention continues to be paid to a possible increase in infections resulting from risk behaviours observed, such as slamming in the context of chemsex sessions.

People experiencing homeless is particularly vulnerable to HCV infection. A study conducted in Catalonia revealed a prevalence of 13.7% in the population under investigation [22]. A number of studies conducted in harm reduction centres have indicated that 30–40% of regular users are homeless [12, 23]. In young injecting drug users attending these centres, a higher prevalence of HCV and its co-infection with HIV was observed among those who are homeless [17]. Within prison settings, 18.5% of cases detected at the time of admission were among individuals experiencing homelessness, thereby underscoring their heightened vulnerability to HCV infection [14].

Catalan prisons, which we could consider as epidemiological observatories due to wide screening coverage, identify some sub-groups or individuals, including large numbers of PWID and migrants, who remain hidden in the community [8]. Infections among those coming from the community amounted to 2.7%, 85% of these were first infections, which were usually related to those detected in the HRCs [14]. At prison’s setting 88% of these detected occur in people with a history or use of injected drugs and 23.7% are previously unknown infections, mainly among the foreign population [24].

## Interventions for the prevention and management of HCV

The strategies implemented in Catalonia for the elimination of HCV have been aligned with WHO recommendations [7], particularly targeting PWID [10]. These strategies are built on decentralised care models operating through drug consumption rooms (DCR) and HRCs through Points of Care [3, 19, 25, 26]. Such facilities have demonstrated efficacy in increasing health system access, reducing transmission through screening, and expanding treatment coverage [8]. Decentralised diagnosis and treatment models ensure a streamlined care continuum, improving treatment access by 57%, cure rates by 41%, reducing loss to follow-up by 41%, and reinfection by 3% compared to conventional approaches [19, 25]. Additional benefits have been observed among those achieving virological cure, including reduced injection frequency, decreased high-risk behaviours, and improved housing conditions [12].

Catalonia has a consolidated harm reduction network for people who use drug (PWUD), comprising more than 60 treatment centres and 20 HRCs, of which 15 include DCR for injection and inhalation. Furthermore, there are more than 700 points of exchange of syringes, which are distributed in pharmacies, primary care and social services [23, 27]. This network offers a wide territorial coverage and comprehensive services, aimed especially at PWID, who represent 41% of clients [28]. This model, in operation for over 30 years, is key to the strategy to eliminate HCV in Catalonia, as it does not require abstinence from treatment, a fact that increases cure rates, reduces transmission (including reinfection) and promotes more equitable access to health care [29].

Despite advances in HCV prevention and care, barriers persist in the community setting, particularly for PWID populations. Individual barriers include being a migrant or a woman, unstable housing, disconnection from health services, and intensive drug use. Systemic challenges include language difficulties and fragmented health and social services [30]. Women who inject drugs face compounded barriers—such as criminalisation, gender-based violence, stigma, and the absence of gender-sensitive services—which hinder access to harm reduction and HCV care [31].

In 2020, a dedicated programme was implemented in the first state residential centre for people experiencing homelessness who also have substance use disorders. However, at the present time, no evaluation data is available. Whilst this facility constitutes a provisional and transient response to the issue of housing insecurity, it furnishes hygienic consumption spaces and incorporates integrated social and healthcare services. Despite its consolidation within public health resources, its capacity remains limited.

In Catalonia, HCV elimination strategies in prisons follow international best practices [32], including universal opt-out screening, accessible and continuous treatment, and the integration of education and harm reduction measures. Current standard measures include OST, Needle exchange programmes (NEP), and condom distribution [6, 33]. Opt-out screening coverage has been achieved at 86%, with sustained virological response rates surpassing 90% of treated cases, supporting the projected elimination of HCV in prisons by 2021 [11]. Although updated data is pending, available evidence suggests that elimination goals have likely been met (set at a prevalence of less than 0.5%) [11], with a reinfection rate of 2 per 100 person-years, mainly among individuals post-release [14].

The viability of universal treatment has been facilitated by administrative support and dedicated prison health teams [34]. Treating people living in prisons has been a strategy with both individual and collective benefits [11, 34, 35]. The liaison nurse role has proven key to ensuring continuity of care upon release [36]. Evidence shows that with appropriate organization and resources, prison-based HCV treatment is not only feasible but also cost-effective [34, 37], offering a model that may be replicated elsewhere. Notably, such interventions have not been implemented in populations with non-custodial sentences, unlike other comparable settings [38]. Although no major internal barriers to HCV elimination have been identified within Catalan prisons, external factors—such as political shifts or funding constraints—could compromise long-term success. The strategy implemented in Cantabria was also a pioneering initiative in the elimination of hepatitis C in prisons [39] within the Spanish territory, illustrating the growing recognition of prison-based treatment strategies.

In Catalonia, the approach to HCV is constrained by a number of limitations, primarily due to the absence of a systematic and homogeneous micro-elimination strategy throughout the territory, particularly among vulnerable populations. The fragmentation of the health system and the weak integration between primary care, addiction units, prison health and hospitals hinder a coordinated response. Furthermore, the absence of universal screening programmes and the over-reliance on individual professional judgment result in disparities in the diagnosis and treatment of patients. The social stigma associated with HCV, particularly its association with drug use, has the effect of limiting access to care, as many people avoid seeking treatment for fear of discrimination. Moreover, the risk of re-infection after successful treatment remains a challenge, particularly in populations with inadequately addressed risk behaviours.

## Recommendations for eliminating HCV among PWIDs in Catalonia

A series of intervention proposals have been formulated with the objective of reinforcing the screening, treatment and monitoring of HCV among PWIDs, with a particular emphasis on vulnerable subpopulations. These proposals also call for the intensification of these tasks within the programmes for reducing damage in community services and prisons in Catalonia.

Integrated care and coordination of services (penitentiary, community and social services) significantly improve adherence to HCV treatment, but also health results (beyond the absence of illness). The most effective interventions include those that simplify diagnostic tests, favour the patients’ commitment to the treatment and promote the participation of health suppliers. From the first point of contact with healthcare services the “cascade of HCV care” needs to be established, with close monitoring that guides individuals’ diagnosis to cure [19, 30]. For this purpose, the first step is to regularly offer rapid tests, even if they report a previous infection, and especially at an HRC [8, 9, 25]. This allows for determining the serological status of PWIDs and initiating treatment early.

Despite efforts to integrate HCV care into services frequented by PWIDs, significantly underdiagnosed subpopulations have been identified [8, 26, 40]. Special attention is needed for vulnerable groups such as migrants, women, youth and homeless individuals [30, 40], as well as for sex workers and people who inject drugs in chemsex contexts (slamming) [20, 41]. A key element of the public health policies should be the promotion of knowledge regarding these sub-populations, with the objective of enhancing the implementation of targeted interventions.

Regarding women, they face multiple vulnerability factors that increase the risk of developing HCV, as well as greater barriers to accessing treatment and preventive measures compared to men [17, 31]. It is essential to address the structural health barriers women face, such as stigma, criminalization, violence, and inadequate resources, to facilitate their access to treatment and harm reduction programs [31]. To achieve this, an intersectional gender approach should be promoted, to address gender inequalities in parallel to those generated by ethnicity and social class [30, 31].

With respect to migrants, administrative improvements that guarantee a rapid recognition of the residence (the basis of the National Health System universality) should be promoted for access to the treatment of HCV [30]. Similarly, health education should involve cultural mediators and community health agents of the target communities, when there are linguistic and cultural barriers [8, 19], as demonstrated for other communicable diseases in a public health context [42, 43]. Furthermore, peer education by previously trained PWIDs must be promoted for greater adaptation and credibility in preventive messages [44].

For individuals participating in chemsex sessions, a comprehensive harm reduction approach is needed. This approach should guarantee access to specialized, interdisciplinary services with expertise in substances, consumption methods, and usage contexts. It should also consider the relationship between these factors and gender socialization and sexual orientation. And their implications for sexually transmitted and bloodborne infections, including HCV [41]. Specifically, surveillance should be conducted through rapid tests at specialised care points for this group, offering screening to those persons whose practices entail a higher risk, although the single-step diagnosis strategy is not recommended, and the referral of cases should be done through established priority circuits [16, 21].

Among people experiencing homelessness, it is recommended that outreach strategies be implemented in order to disseminate information and preventive measures [18]. In a broader sense, policies designed to enhance social factors– including the promotion of Housing First approaches– are imperative to ensure access to HCV treatment [19] and to support treatment adherence and completion [30]. In this context, the involvement of social services and third-sector organizations is crucial.

In general, it is important to intensify preventive measures from the standpoint of harm reduction through the provision of NEP, DCR, and OST, and also pay special attention to the use of stimulants such as cocaine and methamphetamine, so as to promote the stabilisation of PWIDs and guarantee adhesion to HCV treatment [23, 26, 40]. With respect to harm reduction services, it is important to overcome access barriers by making norms more flexible, increasing time coverage, and improving the spaces adapted to the needs of PWIDs, in order to favour more means and opportunities to consume in safe, hygienic conditions [45, 46].

Similarly, peer education approaches are recommended to improve social support, reduce stigma and improve access to HCV treatment among PWIDs [30, 40]. It is also advisable to work on personal barriers, as well as on interpersonal ones with professionals from harm reduction centres, and on systemic ones for greater access to the screening, monitoring and treatment of PWIDs [8, 23, 30].

As for prisons, entering PWIDs continue with HCV infection risk practices, which makes it necessary to prioritise the specific prison programme and interventions [5, 47, 48]. In this sense, universal screening is maintained in order to detect populations with hidden infections and facilitate access to treatment and follow-up [11, 14]. Circuits should be adapted between prison and community, especially between PWID, to make them more effective [30]. The time between diagnosis and treatment should be reduced as much as possible to minimise the risk of patient loss and transmission in cases involving active consumption [35]; for example, with the simplification of the study by non-invasive assessment of hepatic fibrosis [49]. Multidisciplinary work is pivotal in this context, including specialized nurses and family doctors, and even telemedicine when face-to-face attendance lengthens or hinders the process [50].

In order to achieve the desired impact, it is essential to enhance the effectiveness of harm reduction programmes and psychosocial approaches. These have been shown to be pivotal in facilitating stabilisation and promoting adherence to treatment [11, 14, 47]. The coverage of NEP needs to be increased to facilitate hygienic material and to maintain the elimination of intra- prison transmission and reinfection [35, 51, 52]. OST must be promoted in order to reduce risk practices and increase the stabilisation of PWIDs who consume opioids [51, 52].

In addition, it is necessary to promote multidisciplinary health education and advice on adherence to HCV treatment and treatment retention reinforcement following release to the community [51, 53]. More specifically, it is important to promote interventions based on peer education that emphasise working on risk behaviours and promoting change in the beliefs and attitudes towards blood-borne and sexual transmission infections [47].

Finally, it is necessary to guarantee the longitudinal continuity of the care of the persons diagnosed and of the treatment, as well as between prison and the community in order to strengthen adhesion and the cure of HCV [11, 14, 48]. In addition, the integration of the various levels and resources of care– primary care, drug treatment, hospital care and social resources– is imperative in order to reduce the fragmentation of the process. Improvements in primary care must also be implemented for the detection of people with risk factors or HCV and the referral of cases. Professionals are furthermore advised to improve their knowledge on HCV transmission, epidemiology and associated risk factors and to improve the circuits between primary care and services for HCV treatment [16]. Furthermore, there is a necessity to address the issue of destigmatisation by health professionals in order to facilitate the provision of care for PWUD [30, 40].

Overall, both in the community and in prisons, more studies on innovative interventions are required to overcome barriers impeding access to treatment and preventive measures, while considering social determinants of health into account, as well as analysis focused on subpopulations that remain concealed or face greater inequalities in health [15, 17, 26]. In-depth analysis of these specificities can inform the development of preventive and care interventions tailored to the structural and cultural needs of different subpopulations [26]. More specifically, more qualitative studies are required to understand the social determinants of health and the perceptions different subpopulations have related to HCV infection and treatment [30, 52].

## Conclusions

In accordance with the guidelines established by the World Health Organization (WHO), the autonomous community of Catalonia has instituted a comprehensive plan to address and prevent HCV in both community and prison settings. The plan focuses particularly on PWID, and has yielded demonstrable benefits both individually and collectively. The strategies employed include the reinforcement of screening measures, the promotion of universal access to treatment, and the advocacy of harm reduction initiatives, with a tailored approach to specific contexts. Nevertheless, structural and social barriers persist for specific subpopulations, partly due to system fragmentation and limited integration between health and social services.

In order to move towards HCV elimination, greater inter-organizational coordination, effective integration of social and health services, simplification of care pathways, and strengthening of screening are recommended. The training of professionals, the promotion of research aimed at understanding the needs and barriers of those most affected, and the setting of measurable impact targets are also key. In order to enhance prevention, there is a necessity to implement culturally appropriate strategies and participatory approaches, including peer-to-peer work. Within the context of the prison environment, it is imperative to maintain universal screening, adapt care circuits, and promote harm reduction interventions and treatment with OST. A comprehensive, coordinated and people-centred response to drug use in prisons is essentialConsidering the social determinants of health perspective, it is essential that policies and programs are structured to reduce structural inequities and vulnerabilities, thereby promoting equity in both access to prevention, care, treatment and health benefits across all population groups, particularly those most affected [54].

## Data Availability

No datasets were generated or analysed during the current study.

## Abbreviations

DAA: Direct-acting antivirals
DC: Drug centre
DCR: Drug consumption room
HCV: Hepatitis C virus
HIV: Human immunodeficiency virus
HRC: Harm Reduction Centre
MSM: Men who have sex with men
NEP: Needle exchange program
OST: Opioid substitution treatment
PWID: People who inject drugs
PWUD: People who use drugs
WHO: World Health Organization
